# Supplementary material for: Identifying leptospirosis hotspots in Selangor: uncovering climatic connections using remote sensing and developing a predictive model
Source: PeerJ. 2025 Mar 5;13:e18851. doi: 10.7717/peerj.18851 (PMC11890033; doi:10.7717/peerj.18851)
Supplement: Supplemental Information 3 — LGBM code snippets were used during machine learning analysis in the Python 3 software within the Jupyter Notebook platform (Anaconda Navigator environment) to develop the hotspot area predictive model. [file peerj-13-18851-s003.docx]

import numpy as np

import pandas as pd

from sklearn.linear_model import LogisticRegression

from sklearn.model_selection import train_test_split, GridSearchCV, StratifiedKFold, cross_val_score, RepeatedStratifiedKFold

from sklearn.preprocessing import MinMaxScaler, RobustScaler, StandardScaler

from imblearn.pipeline import Pipeline as imbpipeline

from sklearn.pipeline import Pipeline

from sklearn.neighbors import KNeighborsClassifier

from sklearn.svm import SVC

from xgboost.sklearn import XGBClassifier

import xgboost as xgb

import matplotlib.pyplot as plt

import seaborn as sns

from imblearn.over_sampling import SMOTE

from sklearn.model_selection import GridSearchCV

from lightgbm import LGBMClassifier

import lightgbm as lgb

from sklearn.metrics import classification_report, confusion_matrix, roc_curve, roc_auc_score

from sklearn.metrics import precision_score, recall_score, f1_score, roc_auc_score, confusion_matrix

from sklearn.compose import ColumnTransformer

from sklearn.utils import compute_class_weight

from sklearn.utils import class_weight

from sklearn.preprocessing import OneHotEncoder

from collections import Counter

from joblib import dump, load

---------------------------------------

df = pd.read_csv('Test Analysis 1 25-5-24.csv')

---------------------------------------

# Preprocessing

df['Year'] = pd.to_datetime(df['Year'], format='%Y')

df['Month'] = pd.to_datetime(df['Month'], format='%m')

df['month'] = df['Month'].dt.month

df['year'] = df['Year'].dt.year

df.drop(['Year', 'Month'], axis=1, inplace=True)

---------------------------------------

# Feature and target definitions # Prepare for LGBM

num_cols = ['P', 'T', 'month', 'year']

X = df[num_cols]

y = df['H']

---------------------------------------

#Adjust Hyperparameters

n_estimators = [50, 100]

max_depth = [3,4,5, 7]

min_child_weight = [2, 3, 5]

booster = ['gbdt']

base_score = [0.5,0.6]

learning_rate = [0.01, 0.05, 0.1,0.2]

objective = ['binary']

seed = [27]

gamma= [0.7,0.8,0.9]

colsample_bytree=[0.7,0.8,0.9]

subsample=[0.6,0.7,0.8]

reg_alpha = [0.01, 0.05, 0.1]

weights = [0.7, 0.9]

---------------------------------------

#Data split: Training & Cross-validation (0.8), Test (0.2)

X_train, X_test, y_train, y_test = train_test_split(X, y, test_size=0.2, random_state=42)

---------------------------------------

#Preprocessing

preprocessor = ColumnTransformer(

transformers=[

('num', StandardScaler(), num_cols),

],

remainder='passthrough'

)

pipeline = imbpipeline([('smote', SMOTE(random_state=11)),

('scaler', preprocessor),

('classifier', LGBMClassifier())])

stratified_kfold = StratifiedKFold(n_splits=10,

shuffle=True,

random_state=11)

lgbm_params = {'classifier__n_estimators': n_estimators, 'classifier__max_depth': max_depth, 'classifier__learning_rate' : learning_rate, 'classifier__min_child_weight' : min_child_weight,

'classifier__boosting_type' : booster,'smote__sampling_strategy': weights, 'classifier__reg_alpha':reg_alpha}

param_grid = lgbm_params

grid_search = GridSearchCV(estimator=pipeline,

param_grid=param_grid,

scoring='roc_auc',

cv=stratified_kfold,

n_jobs=-1)

---------------------------------------

grid_search.fit(X_train, y_train)

---------------------------------------

end_time = time.time()

print(f'Time taken: {end_time - start_time:.3f} seconds')

---------------------------------------

cv_score = grid_search.best_score_

test_score = grid_search.score(X_test, y_test)

print(f'Cross-validation score: {cv_score}\nTest score: {test_score}')

---------------------------------------

# Get the best estimator from the GridSearchCV object

best_estimator = grid_search.best_estimator_

# Get the predicted probabilities for the test set

y_test_proba = best_estimator.predict_proba(X_test)[:, 1]

# Compute the fpr, tpr, and thresholds for the ROC curve

fpr, tpr, thresholds = roc_curve(y_test, y_test_proba)

# Plot the ROC curve

plt.plot(fpr, tpr, label='ROC curve')

plt.plot([0, 1], [0, 1], 'k--', label='Random guess')

plt.xlabel('False Positive Rate')

plt.ylabel('True Positive Rate')

plt.title('ROC Curve')

# Compute the AUC

auc = roc_auc_score(y_test, y_test_proba)

# Add the AUC score to the graph

plt.annotate(f'AUC = {auc:.4f}', xy=(0.8, 0.2), xycoords='axes fraction')

plt.legend(loc='best')

plt.show()

---------------------------------------

# retrieve the best estimator from the grid search

best_estimator = grid_search.best_estimator_

# extract the XGBClassifier from the pipeline

xgb_clf = best_estimator.named_steps['classifier']

# get the feature importances

importances = xgb_clf.feature_importances_

print(importances)

---------------------------------------

df = df.rename(columns={'P': 'Precipitation','T': 'Land Surface Temperature', 'month': 'Month'})

feature_names = df.drop('H', axis=1).columns

print(len(feature_names))

print(len(importances))

---------------------------------------

# Assign feature names

#feature_names = df.drop('H', axis=1).columns

# create a DataFrame with feature importances and feature names as columns

importance_df = pd.DataFrame(data={'feature_names': feature_names, 'importances': importances})

importance_df.sort_values(by='importances', ascending=False, inplace=True)

feature_importances_timeseries = np.array(importance_df)

# Create a bar chart of feature importances

plt.figure(figsize=(12,6))

plt.bar(x=np.arange(importance_df.shape[0]), height=importance_df['importances'])

plt.xticks(np.arange(importance_df.shape[0]), importance_df['feature_names'], rotation=90) # Key line

plt.xlabel('Features')

plt.ylabel('Importance Score')

plt.title('Feature Importances')

plt.show()

---------------------------------------

# Get the best estimator from the GridSearchCV object

best_estimator = grid_search.best_estimator_

# Get the predicted probabilities for the test set

y_test_proba = best_estimator.predict_proba(X_test)[:, 1]

# Define a list of threshold values to check

thresholds = np.linspace(0.0005, 1, 1000)

#[0.0005, 0.001, 0.01, 0.1, 0.2, 0.3, 0.4, 0.5, 0.6, 0.7, 0.8, 0.9, 1]

# Create empty lists to store the results

sensitivities = []

specificities = []

accuracies = []

precisions = []

recalls = []

f1_scores = []

# Iterate over the threshold values

for threshold in thresholds:

# Modify the predicted probabilities based on the threshold

y_test_pred = [1 if prob >= threshold else 0 for prob in y_test_proba]

# Compute the confusion matrix

conf_matrix = confusion_matrix(y_test, y_test_pred)

# Extract true positives, true negatives, false positives, and false negatives

tp = conf_matrix[1,1]

tn = conf_matrix[0,0]

fp = conf_matrix[0,1]

fn = conf_matrix[1,0]

sensitivity = tp / (tp + fn)

specificity = tn / (tn + fp)

accuracy = (tp + tn) / (tp + tn + fp + fn)

precision = (tp+1) / (tp + fp+1) # Add a small value to both numerator and denominator

recall = sensitivity

f1_score = 2 * (precision * recall) / (precision + recall)

# Append the results to the lists

sensitivities.append(sensitivity)

specificities.append(specificity)

accuracies.append(accuracy)

precisions.append(precision)

recalls.append(recall)

f1_scores.append(f1_score)

# Plot the results

plt.plot(thresholds, sensitivities, label='Sensitivity')

plt.plot(thresholds, specificities, label='Specificity')

plt.plot(thresholds, accuracies, label='Accuracy')

#plt.plot(thresholds, precisions, label='Precision')

#plt.plot(thresholds, f1_scores, label='F1-score')

plt.legend()

plt.xlabel('Threshold')

plt.ylabel('Score')

#plt.title('H')

plt.show()

---------------------------------------

# Set the desired threshold

desired_threshold = 0.5

# Modify the predicted probabilities based on the desired threshold

y_test_pred = [1 if prob >= desired_threshold else 0 for prob in y_test_proba]

# Compute the confusion matrix

conf_matrix = confusion_matrix(y_test, y_test_pred)

# Extract true positives, true negatives, false positives, and false negatives

tp = conf_matrix[1, 1]

tn = conf_matrix[0, 0]

fp = conf_matrix[0, 1]

fn = conf_matrix[1, 0]

# Calculate precision, recall, and F1-score

sensitivity = tp / (tp + fn)

specificity = tn / (tn + fp)

accuracy = (tp + tn) / (tp + tn + fp + fn)

precision = (tp) / (tp + fp)

recall = sensitivity

f1_score = 2 * (precision * recall) / (precision + recall)

print("Sensitivity:", sensitivity)

print("Specificity:", specificity)

print("Accuracy:", accuracy)

print("Precision:", precision)

print("Recall:", recall)

print("F1-score:", f1_score)
