# Supplementary material for: Identifying leptospirosis hotspots in Selangor: uncovering climatic connections using remote sensing and developing a predictive model
Source: PeerJ. 2025 Mar 5;13:e18851. doi: 10.7717/peerj.18851 (PMC11890033; doi:10.7717/peerj.18851)
Supplement: Supplemental Information 5 — LGBM code snippets were used during machine learning analysis in the Python 3 software within the Jupyter Notebook platform (Anaconda Navigator environment) to develop the hotspot area predictive model. [file peerj-13-18851-s005.docx]

import numpy as np

import pandas as pd

from sklearn.linear_model import LogisticRegression

from sklearn.model_selection import train_test_split, GridSearchCV, StratifiedKFold, cross_val_score, RepeatedStratifiedKFold

from sklearn.preprocessing import MinMaxScaler, RobustScaler, StandardScaler

from imblearn.pipeline import Pipeline

from sklearn.neighbors import KNeighborsClassifier

from sklearn.preprocessing import OneHotEncoder

from sklearn.svm import SVC

from xgboost.sklearn import XGBClassifier

import xgboost as xgb

import matplotlib.pyplot as plt

import seaborn as sns

from seaborn import heatmap

from sklearn.metrics import classification_report, confusion_matrix, roc_curve, roc_auc_score

from sklearn.compose import ColumnTransformer

from sklearn.utils import compute_class_weight

from joblib import dump, load

from keras.models import Sequential

from keras.layers import LSTM, Dense

from keras.optimizers import Adam

from sklearn.preprocessing import StandardScaler

from sklearn.pipeline import Pipeline

from imblearn.pipeline import Pipeline as imbpipeline

from imblearn.over_sampling import SMOTE

--------------------------

df = pd.read_csv('Test Analysis 1 25-5-24.csv')

--------------------------

# Preprocessing

df['Year'] = pd.to_datetime(df['Year'], format='%Y')

df['Month'] = pd.to_datetime(df['Month'], format='%m')

df['month'] = df['Month'].dt.month

df['year'] = df['Year'].dt.year

df.drop(['Year', 'Month'], axis=1, inplace=True)

--------------------------

# Feature and target definitions # Prepare for LGBM

num_cols = ['P', 'T', 'month', 'year']

X = df[num_cols]

y = df['H']

--------------------------

X_train, X_test, y_train, y_test = train_test_split(X, y, test_size=0.2, stratify=y, random_state=11)

--------------------------

preprocessor = ColumnTransformer(

transformers=[

('num', StandardScaler(), num_cols),

],

remainder='passthrough'

)

weights = [0.8, 0.9]

pipeline = imbpipeline(steps = [['smote', SMOTE(random_state=11)],

['scaler', preprocessor],

['classifier', SVC(probability=True)]])

param_grid = {'classifier__C':[0.2],

'smote__sampling_strategy': weights,

'classifier__kernel':['rbf'],

'classifier__gamma':[0.03, 1]}

grid_search = GridSearchCV(estimator=pipeline,

param_grid=param_grid,

scoring='roc_auc',

n_jobs=-1)

--------------------------

#Execute Training

import time

start_time = time.time()

--------------------------

grid_search.fit(X_train, y_train)

--------------------------

#To monitor training time

end_time = time.time()

print(f'Time taken: {end_time - start_time:.3f} seconds')

--------------------------

cv_score = grid_search.best_score_

test_score = grid_search.score(X_test, y_test)

print(f'Cross-validation score: {cv_score}\nTest score: {test_score}')

--------------------------

# Get the best estimator from the GridSearchCV object

best_estimator = grid_search.best_estimator_

# Get the predicted probabilities for the test set

y_test_proba = best_estimator.predict_proba(X_test)[:, 1]

# Compute the fpr, tpr, and thresholds for the ROC curve

fpr, tpr, thresholds = roc_curve(y_test, y_test_proba)

# Plot the ROC curve

plt.plot(fpr, tpr, label='ROC curve')

plt.plot([0, 1], [0, 1], 'k--', label='Random guess')

plt.xlabel('False Positive Rate')

plt.ylabel('True Positive Rate')

plt.title('ROC Curve')

# Compute the AUC

auc = roc_auc_score(y_test, y_test_proba)

# Add the AUC score to the graph

plt.annotate(f'AUC = {auc:.4f}', xy=(0.8, 0.2), xycoords='axes fraction')

plt.legend(loc='best')

plt.show()

--------------------------

# Get the best estimator from the GridSearchCV object

best_estimator = grid_search.best_estimator_

# Get the predicted probabilities for the test set

y_test_proba = best_estimator.predict_proba(X_test)[:, 1]

# Define a list of threshold values to check

thresholds = np.linspace(0.0005, 1, 1000)

#[0.0005, 0.001, 0.01, 0.1, 0.2, 0.3, 0.4, 0.5, 0.6, 0.7, 0.8, 0.9, 1]

# Create empty lists to store the results

sensitivities = []

specificities = []

accuracies = []

precisions = []

recalls = []

f1_scores = []

# Iterate over the threshold values

for threshold in thresholds:

# Modify the predicted probabilities based on the threshold

y_test_pred = [1 if prob >= threshold else 0 for prob in y_test_proba]

# Compute the confusion matrix

conf_matrix = confusion_matrix(y_test, y_test_pred)

# Extract true positives, true negatives, false positives, and false negatives

tp = conf_matrix[1,1]

tn = conf_matrix[0,0]

fp = conf_matrix[0,1]

fn = conf_matrix[1,0]

sensitivity = tp / (tp + fn)

specificity = tn / (tn + fp)

accuracy = (tp + tn) / (tp + tn + fp + fn)

precision = (tp+1) / (tp + fp+1) # Add a small value to both numerator and denominator

recall = sensitivity

f1_score = 2 * (precision * recall) / (precision + recall)

# Append the results to the lists

sensitivities.append(sensitivity)

specificities.append(specificity)

accuracies.append(accuracy)

precisions.append(precision)

recalls.append(recall)

f1_scores.append(f1_score)

# Plot the results

plt.plot(thresholds, sensitivities, label='Sensitivity')

plt.plot(thresholds, specificities, label='Specificity')

plt.plot(thresholds, accuracies, label='Accuracy')

#plt.plot(thresholds, precisions, label='Precision')

#plt.plot(thresholds, f1_scores, label='F1-score')

plt.legend()

plt.xlabel('Threshold')

plt.ylabel('Score')

#plt.title('H')

plt.show()

--------------------------

# Set the desired threshold

desired_threshold = 0.5

# Modify the predicted probabilities based on the desired threshold

y_test_pred = [1 if prob >= desired_threshold else 0 for prob in y_test_proba]

# Compute the confusion matrix

conf_matrix = confusion_matrix(y_test, y_test_pred)

# Extract true positives, true negatives, false positives, and false negatives

tp = conf_matrix[1, 1]

tn = conf_matrix[0, 0]

fp = conf_matrix[0, 1]

fn = conf_matrix[1, 0]

# Calculate precision, recall, and F1-score

sensitivity = tp / (tp + fn)

specificity = tn / (tn + fp)

accuracy = (tp + tn) / (tp + tn + fp + fn)

precision = (tp) / (tp + fp)

recall = sensitivity

f1_score = 2 * (precision * recall) / (precision + recall)

print("Sensitivity:", sensitivity)

print("Specificity:", specificity)

print("Accuracy:", accuracy)

print("Precision:", precision)

print("Recall:", recall)

print("F1-score:", f1_score)
