# Supplementary material for: Identifying leptospirosis hotspots in Selangor: uncovering climatic connections using remote sensing and developing a predictive model
Source: PeerJ. 2025 Mar 5;13:e18851. doi: 10.7717/peerj.18851 (PMC11890033; doi:10.7717/peerj.18851)
Supplement: Supplemental Information 6 — Each point data shows the monthly distribution of leptospirosis cases in Selangor from 2011 to 2019. The cases were geocoded according to their coordinates of infection locations. [file peerj-13-18851-s006.docx]

| 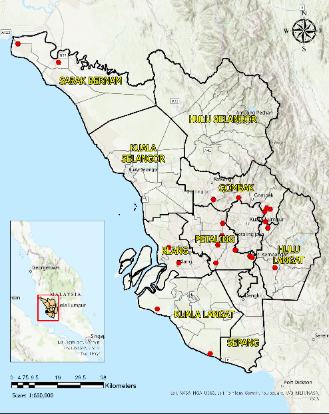January 2011 | 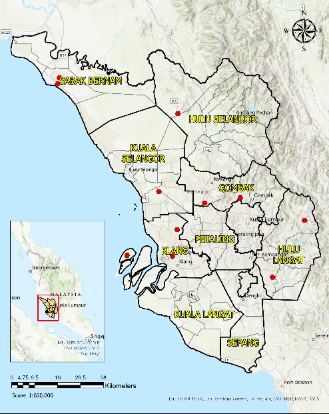February 2011 | 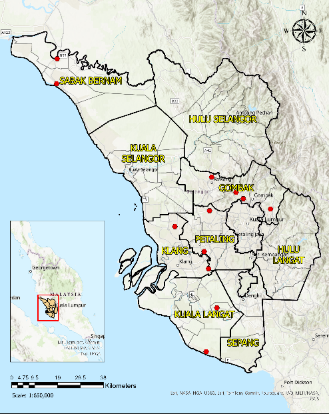March 2011 |
| --- | --- | --- |
| 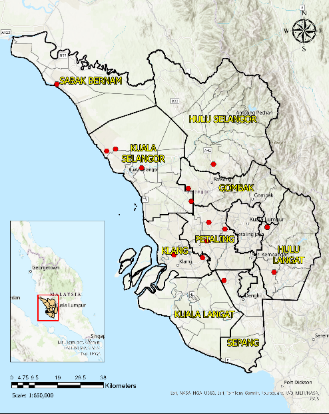April 2011 | 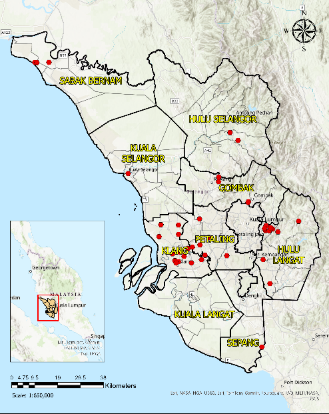  May 2011 | 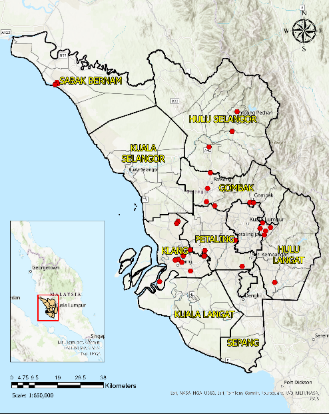  June 2011 |
| 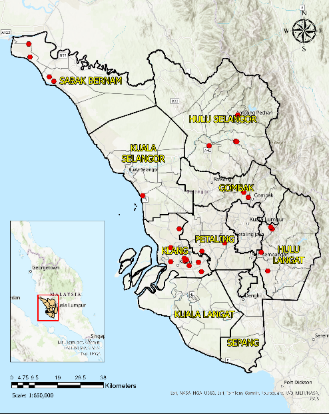  July 2011 | 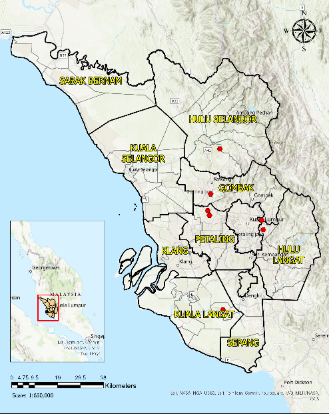August 2011 | 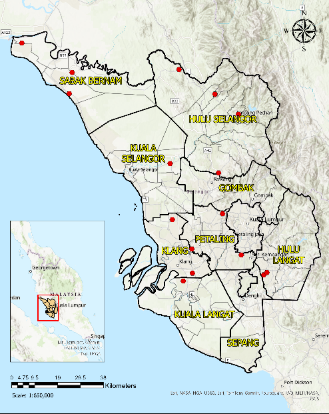September 2011 |
| 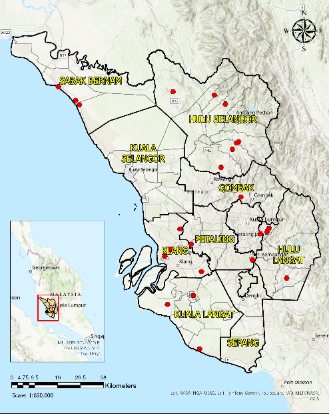October 2011 | 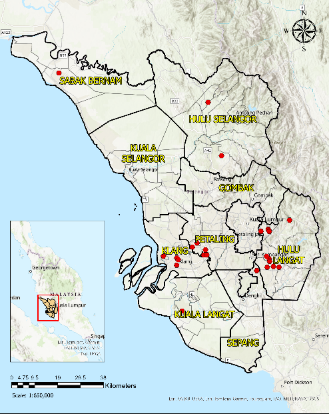 November 2011 | 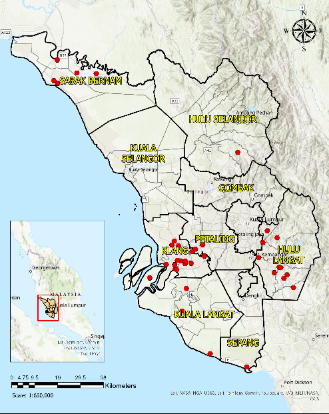 December 2011 |

Figure 1a: Monthly distribution of Leptospirosis in Selangor, 2011

| 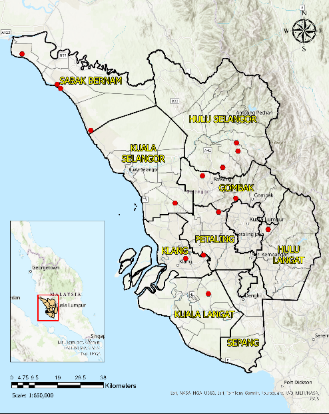January 2012 | 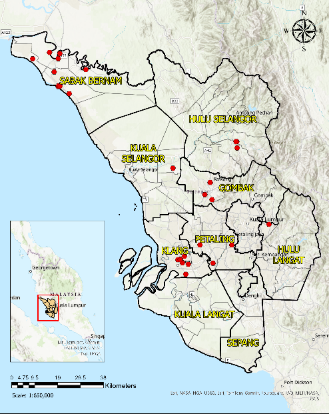 February 2012 | 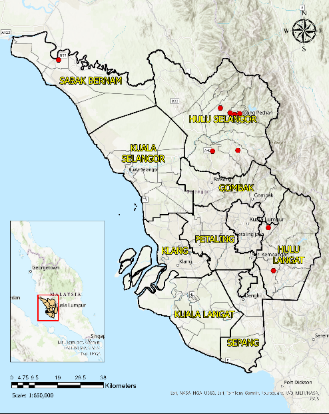 March 2012 |
| --- | --- | --- |
| 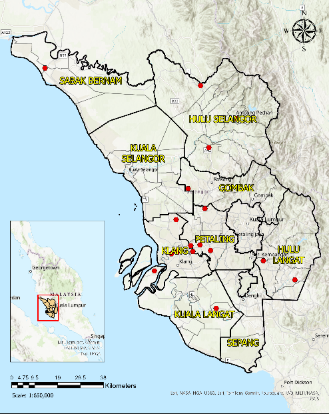 April 2012 | 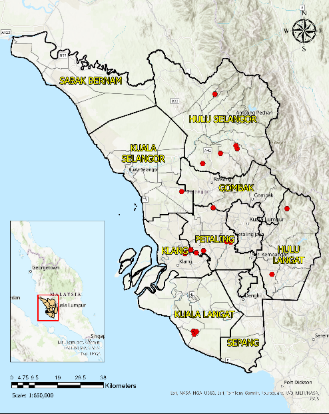 May 2012 | 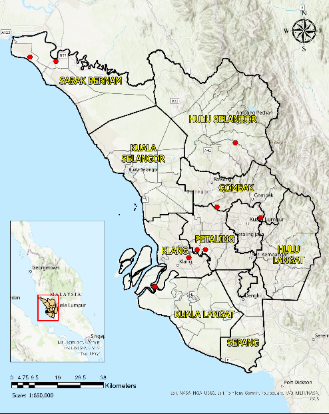 June 2012 |
| 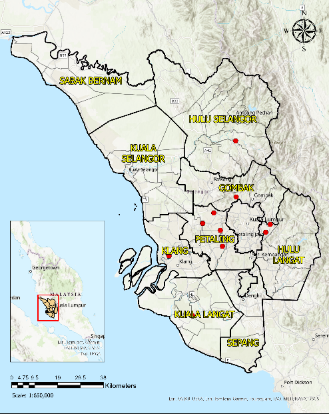 July 2012 | 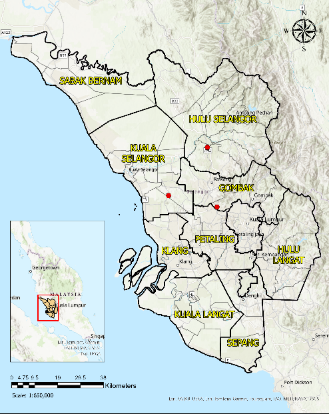 August 2012 | 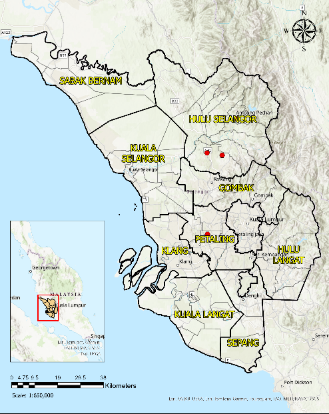 September 2012 |
| 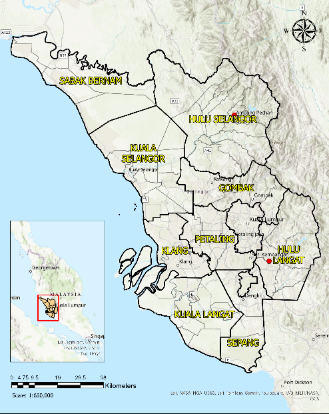 October 2012 | 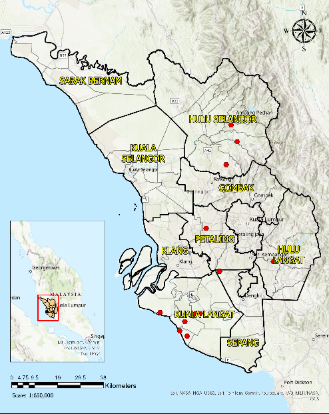 November 2012 | 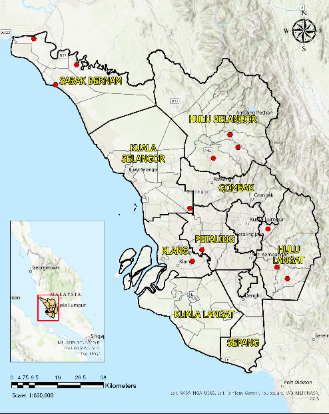 December 2012 |

Figure 1b: Monthly distribution of Leptospirosis in Selangor, 2012

| 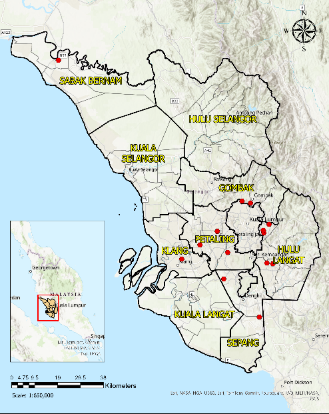January 2013 | 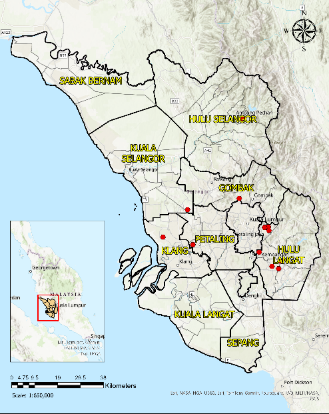 February 2013 | 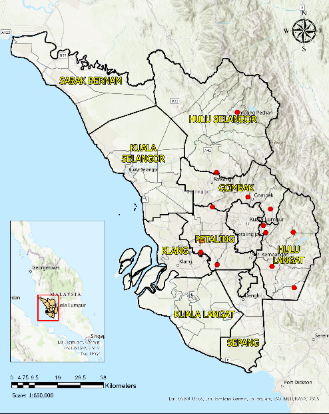 March 2013 |
| --- | --- | --- |
| 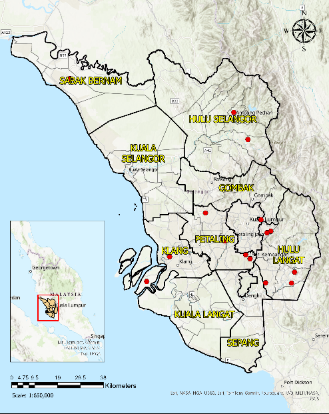 April 2013 | 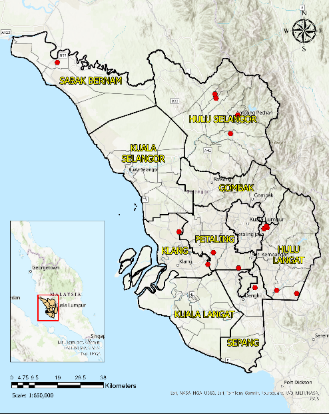 May 2013 | 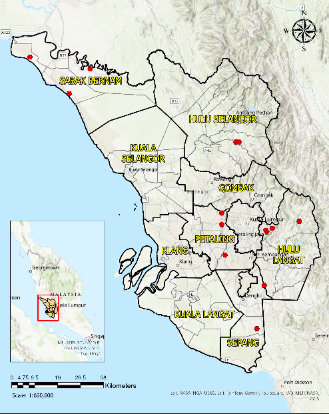 June 2013 |
| 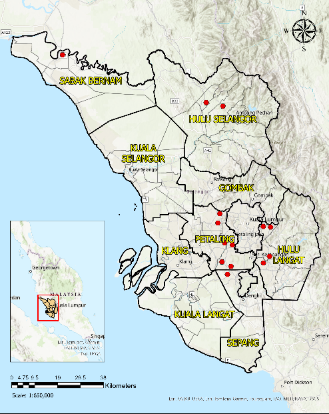 July 2013 | 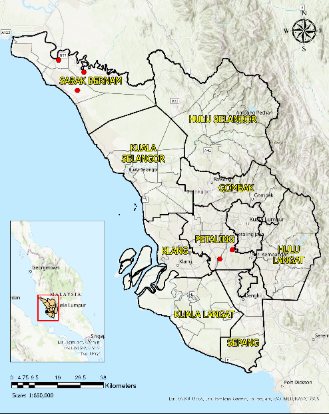 August 2013 | 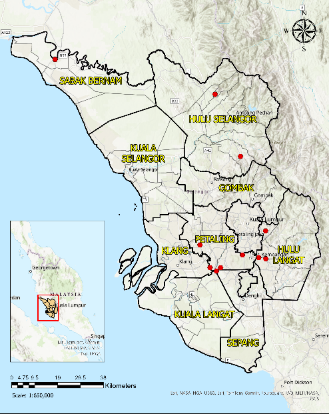 September 2013 |
| 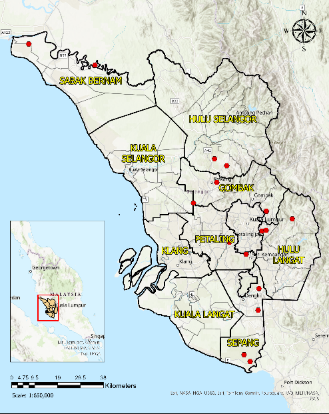 October 2013 | 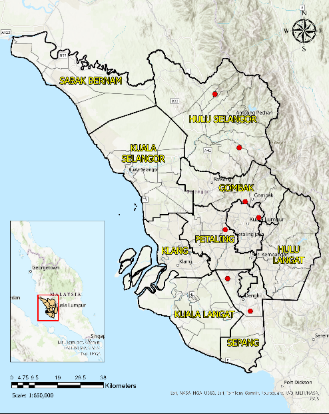 November 2013 | 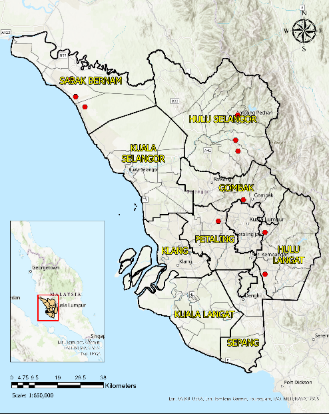 December 2013 |

Figure 1c: Monthly distribution Leptospirosis in Selangor, 2013

| 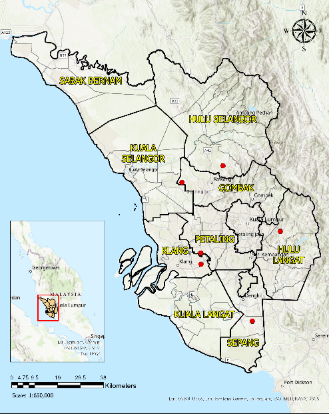 January 2014 | 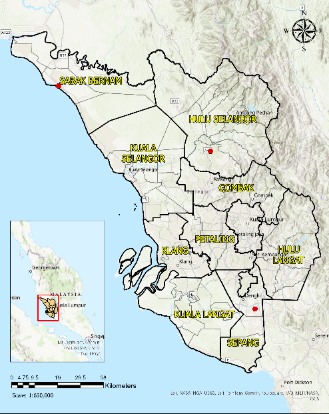 February 2014 | 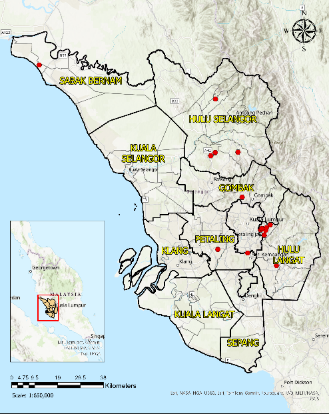 March 2014 |
| --- | --- | --- |
| April 2014 | May 2014 | June 2014 |
| July 2014 | August 2014 | September 2014 |
| October 2014 | November 2014 | December 2014 |

Figure 1d: Monthly distribution of Leptospirosis in Selangor, 2014

| January 2015 | February 2015 | March 2015 |
| --- | --- | --- |
| 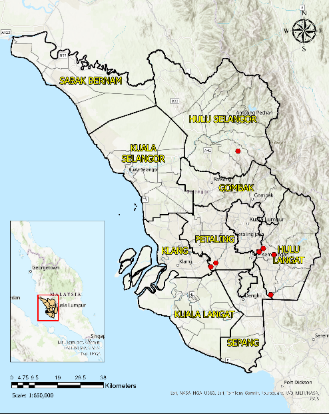 April 2015 | 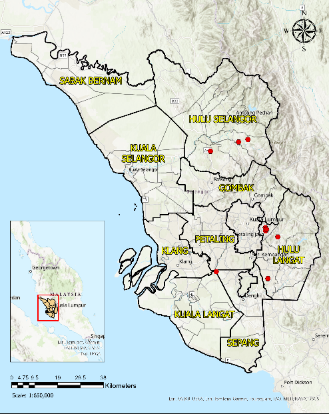 May 2015 | 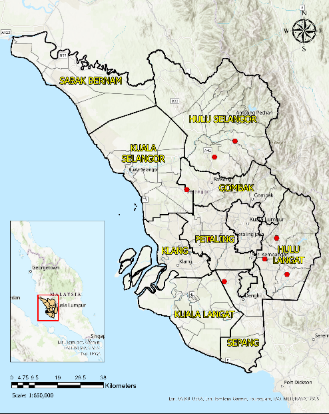 June 2015 |
| 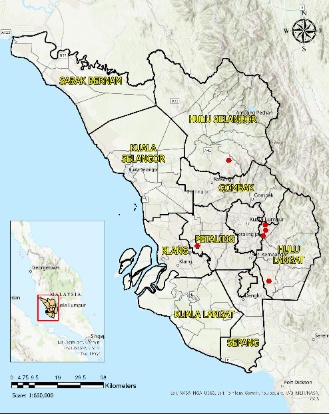 July 2015 | 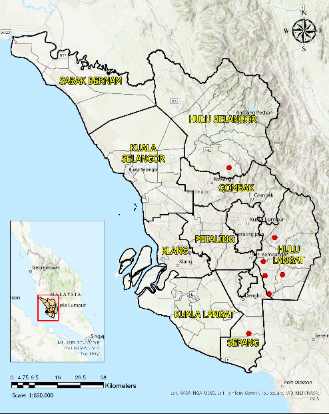 August 2015 | 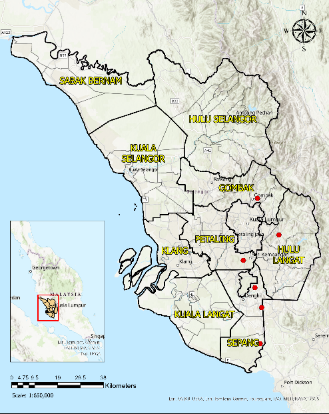 September 2015 |
| 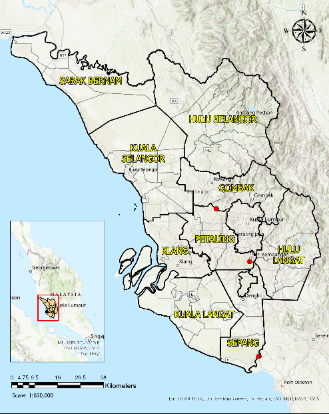 December 2015 |  |  |

Figure 1e: Monthly distribution of Leptospirosis in Selangor, 2015

| 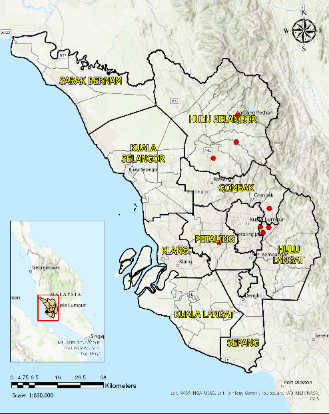 January 2016 | 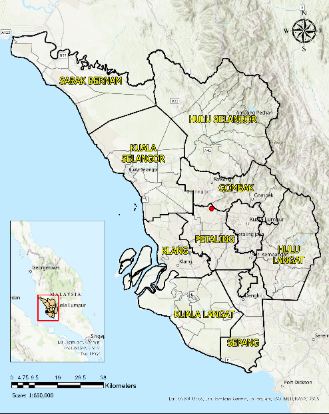 February 2016 | 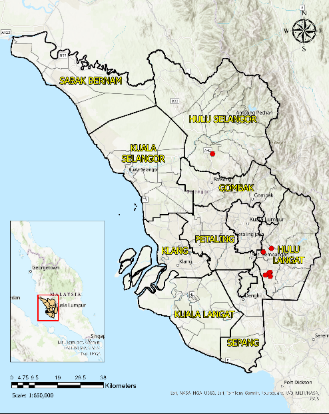 March 2016 |
| --- | --- | --- |
| 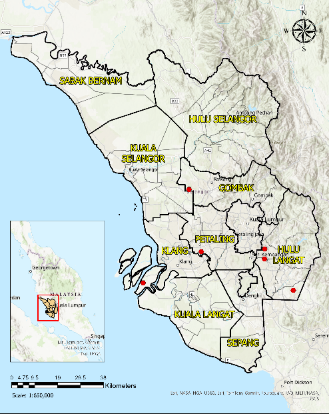 April 2016 | 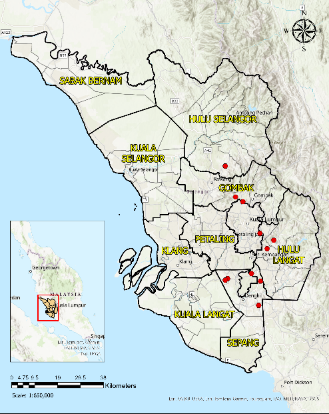 May 2016 | 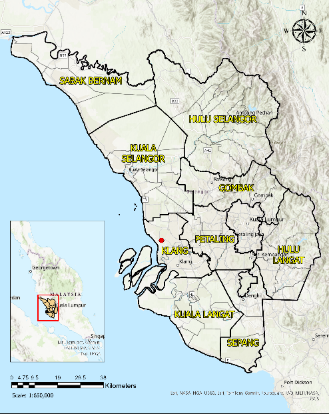 June 2016 |
| 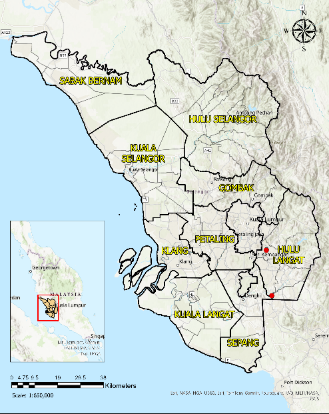 July 2016 | 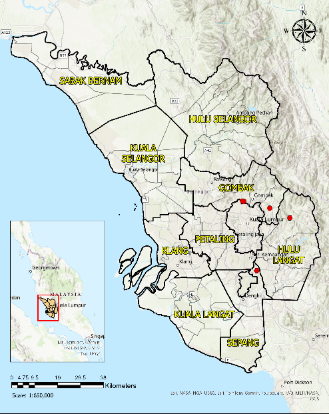 August 2016 | 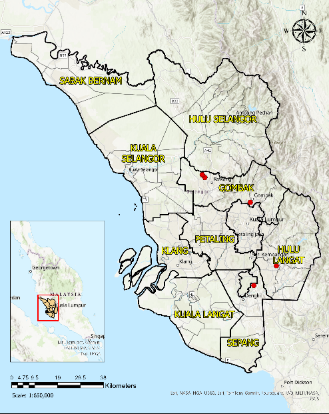 September 2016 |
| 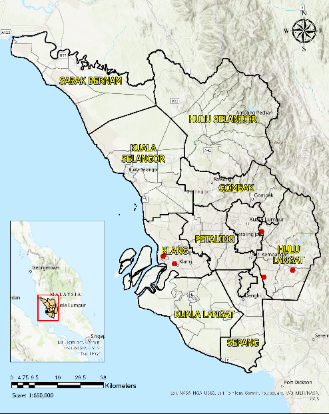 October 2016 | 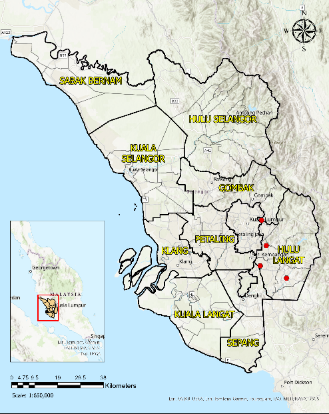 November 2016 | 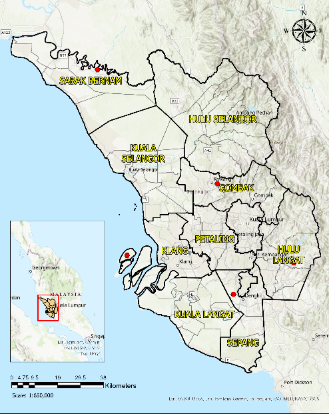 December 2016 |

Figure 1f: Monthly distribution of Leptospirosis in Selangor, 2016

| 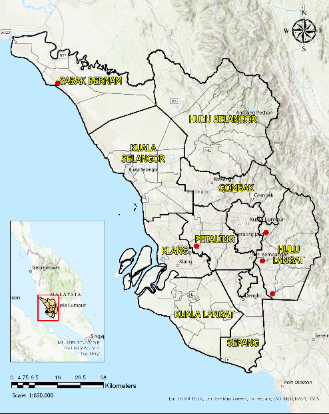 January 2017 | 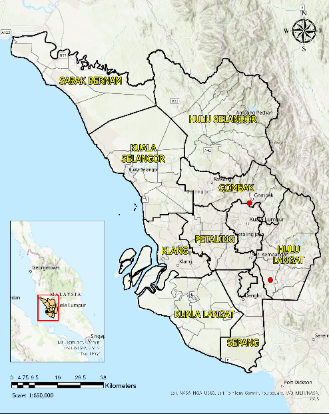 February 2017 | 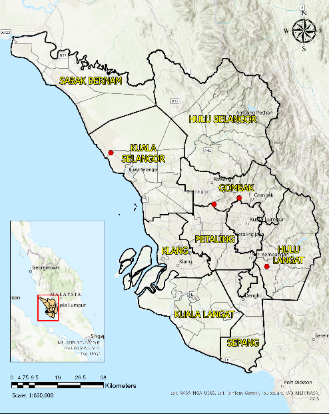 March 2017 |
| --- | --- | --- |
| 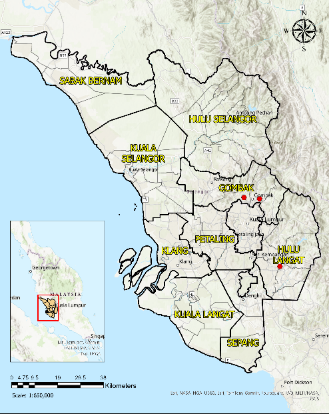 April 2017 | 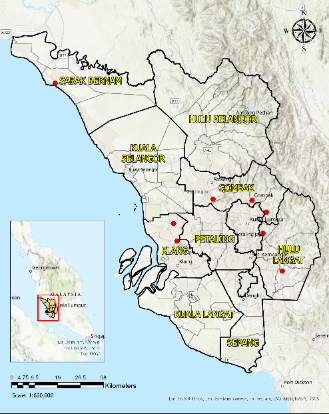 May 2017 | 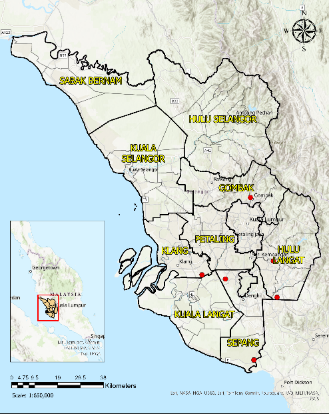 June 2017 |
| 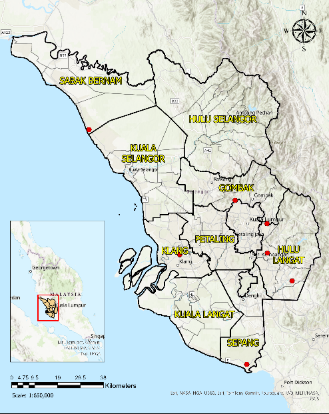 July 2017 | 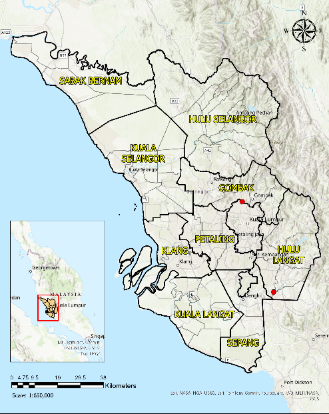 September 2017 | 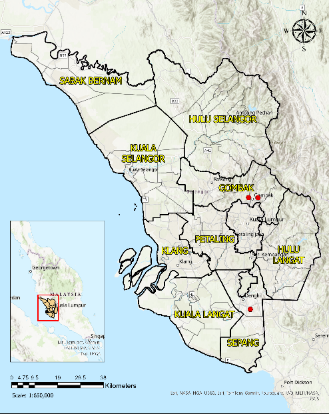 October 2017 |
| 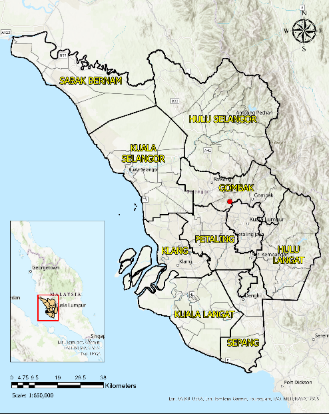 November 2017 |  |  |

Figure 1g: Monthly distribution of Leptospirosis in Selangor, 2017

| 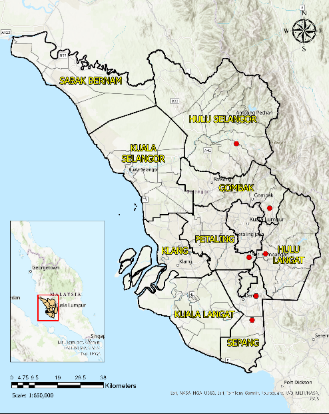 January 2018 | 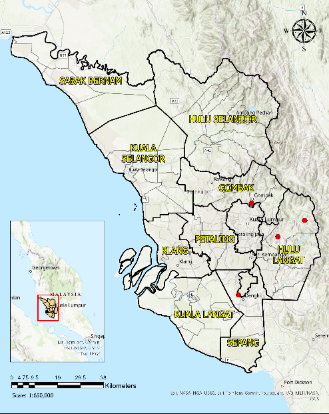 February 2018 | 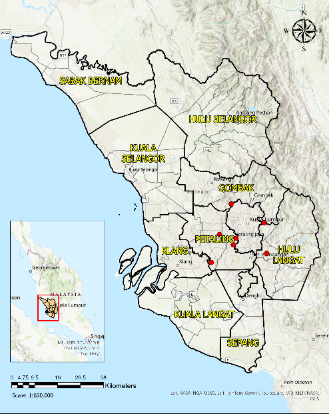 March 2018 |
| --- | --- | --- |
| 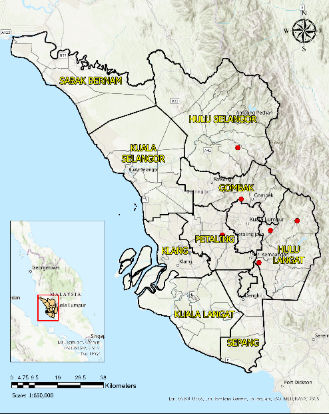 April 2018 | 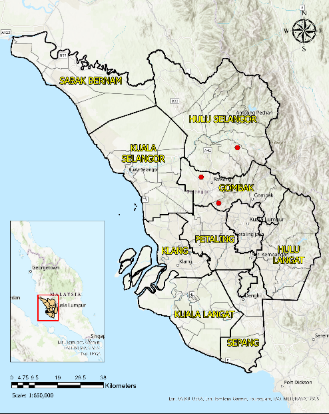 May 2018 | 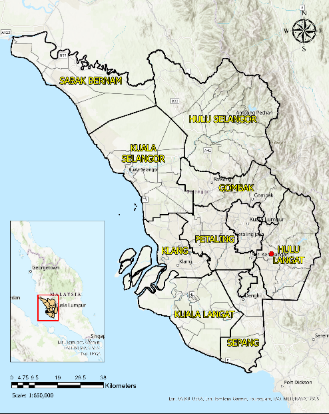 June 2018 |
| 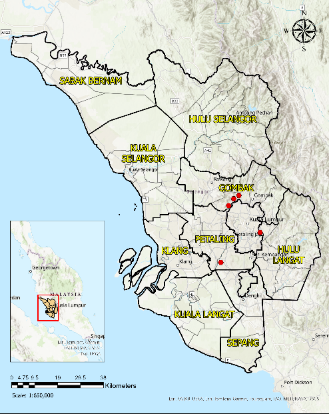 July 2018 | 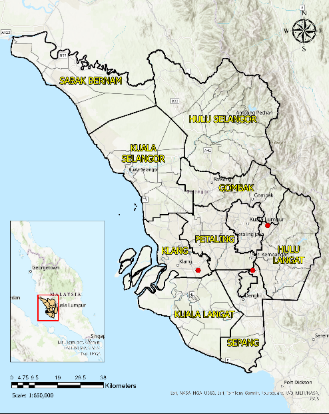 August 2018 | 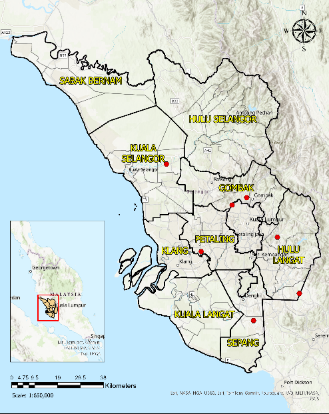  September 2018 |
| 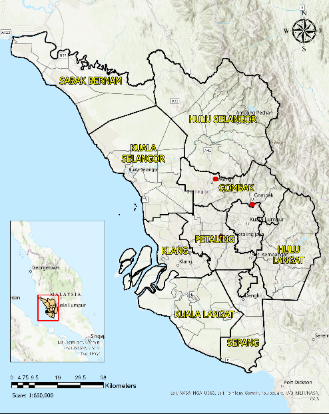 October 2018 | 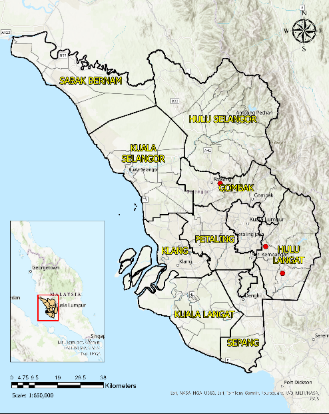 November 2018 | 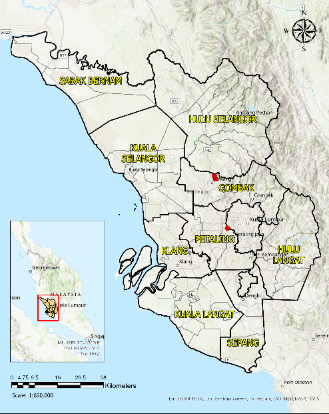 December 2018 |

Figure 1h: Monthly distribution Leptospirosis in Selangor, 2018

| 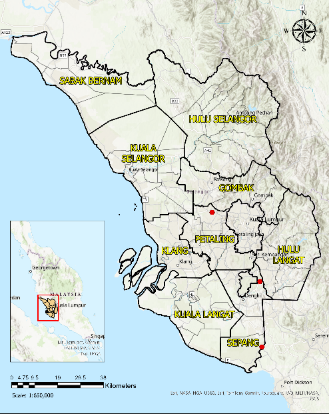 January 2019 | 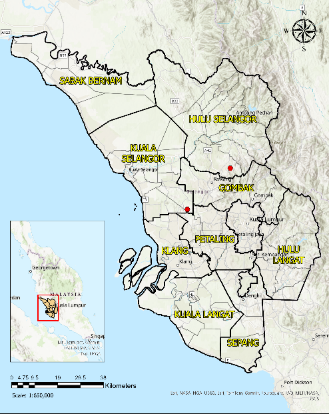 February 2019 | 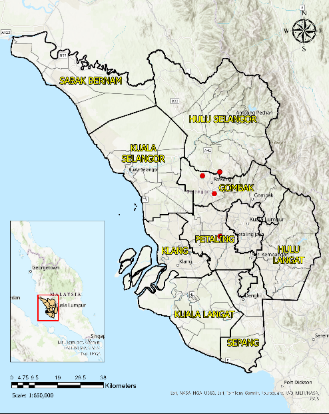 March 2019 |
| --- | --- | --- |
| 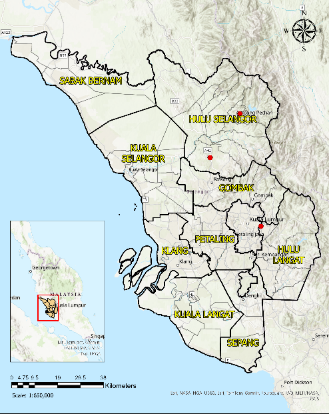 April 2019 | 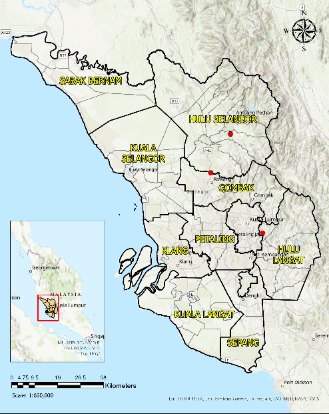 May 2019 | 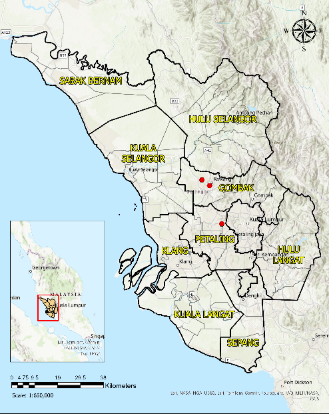 June 2019 |
| 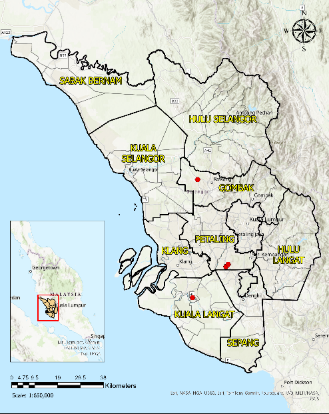 July 2019 | 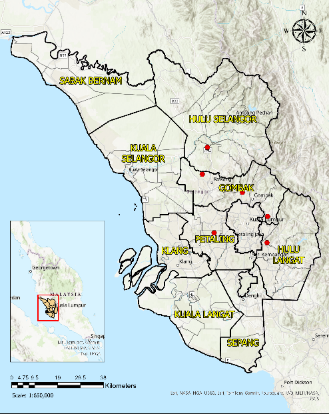 August 2019 | 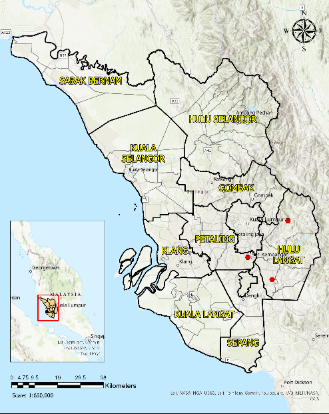  September 2019 |
| 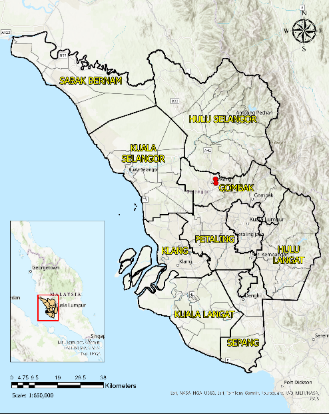 October 2019 | 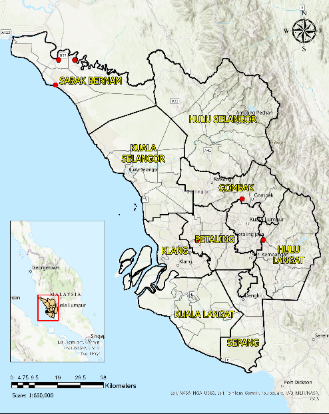 December 2019 |  |

Figure 1i: Monthly distribution of Leptospirosis in Selangor, 2019
