# Supplementary figures and images for: Identifying leptospirosis hotspots in Selangor: uncovering climatic connections using remote sensing and developing a predictive model
Source: PeerJ. 2025 Mar 5;13:e18851. doi: 10.7717/peerj.18851 (PMC11890033; doi:10.7717/peerj.18851)

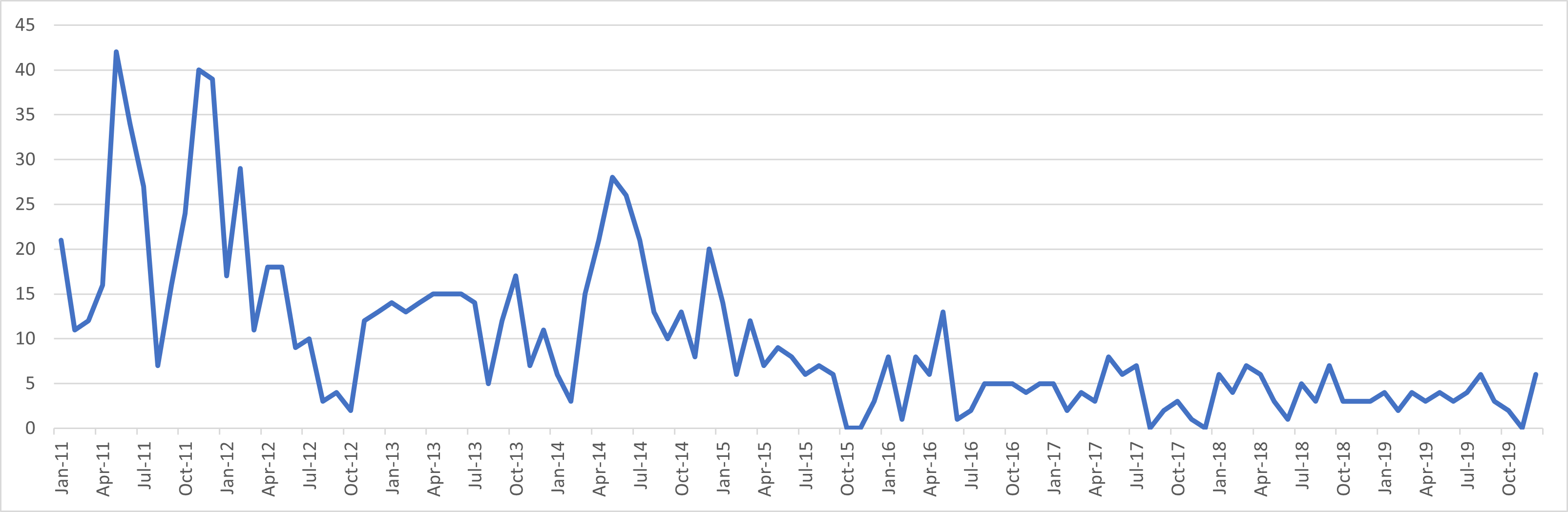

Supplement: Supplemental Information 7 — The graph shows the total monthly leptospirosis cases in Selangor from 2011 to 2019. The peak incidence of leptospirosis occurred between the beginning of 2011 and the first quarter of 2012, followed by a decline until the beginning of 2014. Subsequently, a resurgence of cases occurred in 2014, which declined progressively until 2019. [file peerj-13-18851-s007.png]
